# Responding to medetomidine: clinical and public health needs

**DOI:** 10.1016/j.lana.2025.101053

**Published:** 2025-03-06

**Authors:** David T. Zhu, Joseph J. Palamar

**Affiliations:** a Medical Scientist Training Program, School of Medicine, Virginia Commonwealth University, Richmond, VA, USA; b Department of Population Health, NYU Grossman School of Medicine, New York, NY, USA

The emergence of medetomidine as a novel adulterant in the U.S. illicit drug supply signals a troubling development in the escalating polysubstance crisis. Medetomidine, an α2-adrenergic receptor agonist approved exclusively for veterinary use, has increasingly been implicated in illicit fentanyl-related overdoses. Its high potency—200- to 300-fold greater than xylazine—and rapid geographic spread underscore the need for heightened clinical vigilance and coordinated interventions.^1^

First identified in Maryland's illicit opioid supply in July 2022, medetomidine was frequently co-adulterated with xylazine.^1^^,^^2^ By mid-2023, it had spread to Missouri, Colorado, Pennsylvania, and California, and by early 2024, it was linked to overdose clusters in Philadelphia, Pittsburgh, and Chicago.^1^^,^^2^ This trajectory mirrors the early epicenters of the xylazine overdose crisis—Maryland and Philadelphia—which may serve as bellwethers for emerging drug trends.^1^, ^2^, ^3^ These patterns emphasize the importance of applying lessons from the nation's response to xylazine to address medetomidine's growing threat.

## Clinical manifestations and management

Medetomidine toxicity resembles xylazine toxicity but is often more severe and prolonged due to its higher potency.^1^ Patients typically exhibit profound sedation, bradycardia, hypotension, and central nervous system depression, which may persist longer than symptoms from fentanyl or xylazine alone.^1^^,^^4^^,^^5^ Clinicians should maintain a high index of suspicion in cases of prolonged sedation despite naloxone administration.

Emergency departments and clinics should adapt protocols to address the sedative and cardiovascular effects of medetomidine, particularly respiratory depression and hemodynamic instability. Preparedness for advanced airway management, ventilatory support, and vasopressor therapy is essential. Clonidine, which has shown efficacy in managing dexmedetomidine withdrawal, may be considered for hemodynamically stable patients with persistent symptoms despite receiving opioid agonist therapy.^4^ While naloxone does not directly reverse medetomidine toxicity, as it is not an opioid, it should still be administered in suspected overdoses due to its frequent co-adulteration with fentanyl.^1^

Xylazine injection use has also been linked to necrotic skin ulcers.^3^ Although medetomidine has not been directly associated with such lesions, its frequent co-occurrence with xylazine and vasoconstrictive effects raise concerns about its impact on wound healing.^4^ Further research is needed to differentiate the clinical effects of xylazine and medetomidine overdoses, informing evidence-based treatment protocols.

## Epidemiological surveillance and drug testing

Proactive epidemiological surveillance is needed to identify emerging overdose trends and issue timely public health alerts. Strengthening collaborations among healthcare providers, coroners, toxicologists, law enforcement agencies, and harm reduction organizations is critical to this effort. The National Drug Early Warning System (NDEWS) exemplifies this approach, with its 17 sentinel surveillance sites providing monthly reports to its coordinating center on regional drug trends and enhancing monitoring of novel adulterants like medetomidine.^6^ Through bidirectional information sharing among its network partners, NDEWS offers a scalable model for integrating diverse data streams and coordinating responses to emerging drug threats.

Forensic and toxicology networks are also crucial to advancing monitoring efforts. Notably, the Center for Forensic Science Research and Education, the Drug Enforcement Administration Toxicology Testing Program, and the ToxIC Fentalog Study Group have already begun to systematically identify medetomidine in toxicology specimens and drug products in select U.S. cities. These efforts represent a critical step toward monitoring geographical trends in medetomidine overdoses, particularly those co-involving fentanyl and xylazine.

However, medetomidine is not included in standard toxicology panels, underscoring the need for expanded early detection strategies in clinical and harm reduction settings. Point-of-care test strips for medetomidine offer a practical solution, with BTNX Inc. test strips reliably detecting medetomidine at concentrations above 1000 ng/mL.^7^ These test strips should be distributed in regions with identified medetomidine overdoses to enhance real-time surveillance. Federal agencies, including the Substance Abuse and Mental Health Services Administration, have endorsed fentanyl and xylazine test strips, setting a precedent for similar strategies for medetomidine. Although expensive, confirmatory testing with liquid chromatography–mass spectrometry remains essential for accurately characterizing medetomidine-involved polysubstance overdoses.

## Public health and research needs

A coordinated policy response is essential to expand drug testing and treatment access. New York's public health response to the xylazine overdose crisis offers valuable lessons, particularly through partnerships with organizations like NY MATTERS, which provided free xylazine and fentanyl test strips to healthcare providers and the public, facilitating early detection in illicit drug supplies.^8^ Concurrently, the New York State Office of Addiction Services and Supports implemented training programs for clinicians and outreach workers to manage xylazine-involved overdoses and complications such as skin ulcers.^8^ Supported by opioid settlement funds, harm reduction services also integrated drug-checking and wound care within low-barrier settings like syringe exchange programs and supervised injection sites. This multifaceted model could be adapted to mitigate the medetomidine overdose crisis by prioritizing early detection, treatment, and supportive services.

Future epidemiological studies should prioritize geospatial mapping of medetomidine overdose hotspots and real-time data dissemination to inform targeted public health interventions. Clinical research is needed to develop evidence-based treatment protocols, such as evaluating pharmacotherapies like atipamezole, an α2-adrenergic antagonist shown to reverse medetomidine toxicity in veterinary practice.^5^^,^^9^ Concurrently, biomedical studies are needed to characterize medetomidine's pharmacokinetics and interactions with co-adulterants like fentanyl, xylazine, and nitazenes, which may complicate overdose presentation and treatment.^2^

The burgeoning medetomidine overdose crisis demands coordinated actions from clinicians, public health agencies, and policymakers. The tools for an effective response are within reach: developing medetomidine-specific clinical guidelines, expanding epidemiological surveillance, and fostering multi-sectoral collaborations. However, their success hinges upon our collective resolve to implement them. At this critical juncture, we have an opportunity to act proactively—the question is whether we will act with the urgency the crisis demands.

## Contributors

All authors were involved in the conceptualization, writing, and revision of the manuscript.

## Data sharing statement

Not applicable.

## Declaration of interests

Joseph J Palamar reported multiple research grants from the National Institutes of Health/National Institute on Drug Abuse (NIH/NIDA) (U01DA051126, R01DA060362). Joseph J Palamar reported receiving consulting fees as a paid consultant for a two-day meeting in 2024 with the Washington–Baltimore High Intensity Drug Trafficking Areas program. Joseph J Palamar reported receiving payment or honoraria for lectures, presentations, serving on thesis committees, and other educational events from the University of Florida, Rutgers University, University of Southern California, Arizona State University, University of Queensland (Australia), and Elsevier. Joseph J Palamar reported serving on multiple Elsevier editorial boards. Joseph J Palamar reported receiving support for attending meetings and travel from the University of Florida, NIH/NIDA, the Reagan–Udall Foundation for the Food and Drug Administration (FDA), and the American College of Neuropsychopharmacology. David T Zhu reported no disclosures.
